# Physical Processes during the Formation of Silicon-Lithium p-i-n Structures Using Double-Sided Diffusion and Drift Methods

**DOI:** 10.3390/ma14185174

**Published:** 2021-09-09

**Authors:** Ahmet Saymbetov, Ramizulla Muminov, Nursultan Japashov, Yorkin Toshmurodov, Madiyar Nurgaliyev, Nursultan Koshkarbay, Nurzhigit Kuttybay, Batyrbek Zholamanov, Zhang Jing

**Affiliations:** 1 Faculty of Physics and Technology, Al-Farabi Kazakh National University, Almaty 050040, Kazakhstan; nursultan.dzhapashov@kaznu.kz (N.J.); madiyar.nurgaliyev@kaznu.kz (M.N.); koshkarbay_nursultan@live.kaznu.kz (N.K.); nurzhigit.kuttybyy@kaznu.kz (N.K.); zholamanov_batyrbek1@live.kaznu.kz (B.Z.); chzhan_tszin5@live.kaznu.kz (Z.J.); 2 Physical-Technical Institute, Uzbekistan Academy of Sciences, Tashkent 100084, Uzbekistan; detector@uzsci.net; 3 Tashkent Institute of Irrigation and Agricultural Mechanization Engineers, Tashkent 100000, Uzbekistan; y.toshmurodov@tiiame.uz

**Keywords:** drift of Li-ions, Si(Li) structure, p-i-n detectors, double-sided drift

## Abstract

In this paper, we described a method of double-sided diffusion and drift of lithium-ions into monocrystalline silicon for the formation of the large-sized, p-i-n structured Si(Li) radiation detectors. The p-i-n structure is a p-n junction with a doped region, where the “i-region” is between the n and the p layers. A well-defined i-region is usually associated with p or n layers with high resistivities. The p-i-n structure is mostly used in diodes and in some types of semiconductor radiation detectors. The uniqueness of this method is that, in this method, the processes of diffusion and drift of lithium-ions, which are the main processes in the formation of Si(Li) p-i-n structures, are produced from both flat sides of cylindrical-shaped monocrystalline silicon, at optimal temperature (T = 420 °C) conditions of diffusion, and subsequently, with synchronous supply of temperature (from 55 to 100 °C) and reverse bias voltage (from 70 to 300 V) during drift of lithium-ions into silicon. Thus, shortening the manufacturing time of the detector and providing a more uniform distribution of lithium-ions in the crystal volume. Since, at present, the development of manufacturing of large-sized Si(Li) detectors is hindered due to difficulties in obtaining a uniformly compensated large area and time-consuming manufacturing process, the proposed method may open up new possibilities in detector manufacturing.

## 1. Introduction

There are different types [1,2,3,4] and manufacturing technologies [5,6,7] of Si(Li) p-i-n detectors. For detectors with a large sensitive area, the method of p-type silicon compensation by lithium-ions remains as one of the effective methods [8,9]. This method consists of the following steps: obtaining and processing of appropriate monocrystalline silicon, diffusion of Li atoms into monocrystalline Si to the pre-determined depth, drift of Li-ions into Si and metal contact application to the obtained sample. One of the main processes listed above is the formation of the p-i-n region.

The method of lithium-ion drift for p-i-n formation was originally proposed by Pell [10]. This method found wide application in the production of semiconductor nuclear radiation detectors. Pell proved that if the reverse bias voltage is applied to the p-n junction with sufficient temperature to excite the donor or acceptor ions, then the ions will drift in the electric field to create intrinsic regions between the p-n junctions. Immediately after the pioneering work of Pell, Lechrer and Reis’s [11] work was published on the investigation of the motion of lithium-ions in the region of the p-n junction under reverse bias. Lechrer and Reis derived equations which describe the distribution of lithium-ions in the inner part of the crystal depending on time. Then, Pell’s theory was considered by Gibbon and Iredal [12], with a more precise assumption to real detecting systems. Here, they considered the accuracy of compensation for acceptor impurities in semiconductors. It was also shown that lithium-ion drift technology depends on the distribution of acceptors in the initial crystal. It is said that exact compensation is achieved only if the supplied drift field is an orthogonal with flux of the acceptor impurity. Later, in 1969, Lauber [13] stated that the high quality of compensation in the inner part of the crystal mainly depends on the thermally generated electron–hole pairs that were separated by the reverse bias voltage applied during the drift.

Despite the fact that lithium drift technology has been the subject of research for such a long time, Si(Li) detectors still remain a relevant research object. In recent work [14], a method and hardware implementation of a segmented and nonsegmental lithium drift detector operating at room temperature was developed. Additionally, in [15], the authors have developed a novel type of n+-i- n-p large-area silicon microstrip sensors for very high-radiation environments. Si(Li) p-i-n detectors were also successfully used for the CBERS project in [16]. These detectors have opened up new possibilities for registering gamma- and X-rays.

Despite a number of advantages of semiconductor spectrometry, further development in this field is hampered by technological difficulties of obtaining Si(Li) p-i-n structured detectors, which have a bigger sensitive area and better energy resolution. Manufacturing Si(Li) detectors with a sensitive diameter of more than 6 mm is challenging, because of the increased leakage of current and capacitance. Besides, one of the difficult and very long processes in the development of Si(Li) p-i-n nuclear radiation detectors is the formation of the intrinsic region (i-region) by the drift method. Therefore, to create a sensitive area of the detector, with a thickness of about 4 mm and with diameter up to 5 mm, takes months. In addition, when the i-region is formed in the crystal, an inhomogeneous distribution of lithium-ions arises. Lithium-ions move in the field of applied reverse bias along the interstices of silicon atoms from the surface part into the depth of the crystal; when the electric field weakens, particles encounter different obstacles (defects, complexes of various impurities in the crystal, etc.) and change direction of distribution [17,18,19]. Therefore, when the i-region is formed in the crystal, an inhomogeneous distribution of lithium-ions is caused. Double-sided drift technology can help to avoid this problem: in penetration of lithium-ions in silicon, the lithium-ions are distributed from both sides, the front and the back flat surface of the crystal toward the center of the crystal. As the process is double-sided, the penetration path of lithium-ions is halved, accordingly, the distribution will be more uniform. Another basic factor in the formation of the compensated area is the correct choice of the temperature regime during the drift process [20,21]. To prevent the negative effects associated with the heating of the crystal above the permissible value due to the passage of a large current through the structure, the drift mode must be selected, taking into account the heat balance conditions, according to which the amount of heat released (according to the Joule-Lenz law) must be balanced with the heat removed.

Previously, in [22], our research group briefly reported the experimental results of manufacturing large-sized Si(Li) detector structures with a new method of double-sided diffusion and drift of lithium-ions into monocrystalline silicon. In this paper, we describe the manufacturing procedure of large-sized Si(Li) p-i-n detectors in detail, with optimal regimes of experimental technique and theoretical assumptions of the formulation of p-i-n regions in large-sized monocrystalline silicon obtained by the double-sided method. The aim of the work is to explain the new technology of obtaining Si(Li) p-i-n detectors and emphasize the advantages of the method. At the end of this paper, the electrophysical characteristics of Si(Li) p-i-n detectors are shown, which were obtained experimentally by the proposed method.

## 2. Materials and Methods

To study the formation of a Si(Li) p-i-n structure, first, it is necessary to study the process of double-sided diffusion of lithium atoms into single-crystal silicon for the given parameters: the concentration of donor charge carriers of monocrystalline silicon *N*_0_ = 5 × 10^17^, where lithium atoms diffuse to a depth of 300 μm from two flat surfaces of a silicon wafer. To solve this problem, both numerical and analytical methods for solving diffusion problems were considered. For diffusion, we set a boundary value problem known in mathematical physics with the initial conditions *N*(0, *t*) = *N*(*l*, *t*) = *Ns*, where *N*(0, *t*) are the values of the lithium concentration at the initial moment of time on one side and *N*(*l*, *t*) on the other side of the crystal. This all equates to the total concentration of lithium *Ns*. After writing the main diffusion equation for boundary value problems based on the Rice theory, the effect of the oxygen complex on the effective diffusion coefficient of lithium was studied. To determine the temperature–time modes of diffusion, it is necessary to solve the diffusion equation under the conditions of boundary value problems, taking into account the high concentration of the Li-O complex and the specified boundary conditions. The distribution of lithium symmetrically relative to the center from two plane surfaces of the crystal using double-sided drift was also investigated by analytical and numerical methods. This helps us to clearly understand the physical processes occurring during the drift and select the optimal mode for the drift of lithium-ions into the silicon crystal.

As initial material for the detectors, the dislocation-free monocrystalline cylindrical silicon crystal of the p-type, obtained by the float-zone method (with diameter 110 mm, thickness 4–5 mm, resistivity *ρ* = 1000–5000 Ohm∙cm and with lifetime *τ* ≥ 500 μs), and silicon crystal of the p-type (with a diameter of 110 mm, with a resistivity *ρ* = 10–12 Ohm∙cm, lifetime *τ* ≥ 50 μs, grown in an argon atmosphere), obtained by the Czochralski method, were used. After manufacturing, the electro-physical characteristics of the detectors obtained by two types of silicon were compared.

**Figure 1:**
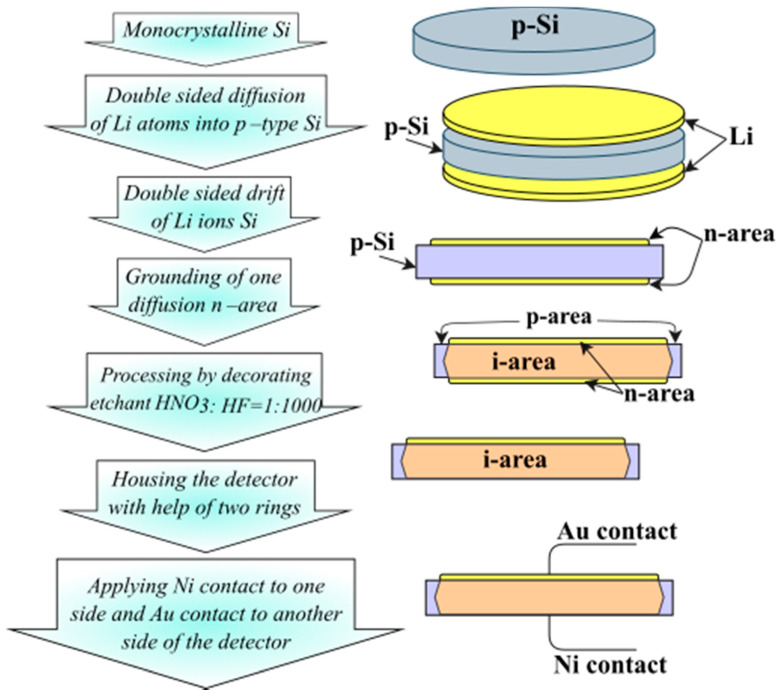
Schematic of manufacturing large-sized detectors, with double-sided diffusion and drift of lithium-ions, into monocrystalline silicon technology.

In the proposed method, lithium is diffused from both surfaces to a predetermined depth (300 μm) sufficient for providing the necessary compensation of the initial acceptor impurity in the required volume to pre-prepared samples of silicon under temperature *t* = 400 °C during *t* = 4 min in a vacuum of *p* = 10^–5^ mmHg. After the diffusion process, by applying a reverse bias voltage and a sufficient temperature from 55 to 110 °C and voltage values from 70 to 300 V, a drift from two flat surfaces to a cylindrical silicon wafer is carried out. The drift completion moment is fixed by a sharp increase of inverse current. As the drift process finishes (i.e., joining of two counter fronts of lithium drift), one of the diffusion n-areas is grounded off to the depth determined by its degradation during the drift process (Figure 1).

## 3. Diffusion of Li Atoms into Si

Monocrystalline Si grown by the Czochralski method is characterized by a pronounced defectiveness of the crystal structure. This is due to the large volume of Li-O complexes in the body of the crystal, which promotes the generation of thermal donors in the crystal, as well as the formation of dipole structures in the places of accumulation of acceptor impurity, which significantly changes the nature of diffusion and drift of lithium-ions [23]. However, the presence of Li-O complexes ensures the stability of Si(Li) p-i-n+ detector structures [24,25]. To solve the problem of diffusion of Li atoms into monocrystalline silicon with a high content of Li-O complexes, the following relation is valid, for the lithium-ion flux:

(1) $$J=-D_{0}\frac{\partial \left(N_{Li}-p\right)}{\partial x}$$

where *D*_0_ is the diffusion coefficient of lithium into pure silicon without oxygen complexes, and $N_{Li}$ is the lithium concentration.

Under the thermodynamic equilibrium of free and connected complexes of lithium from the law of effective masses, we have:

(2) $$\frac{p}{\left(N_{O_{2}}-p\right)\cdot \left(N_{Li}-p\right)}=k\left(T\right)$$

where *k*(*T*) is the equilibrium constant, depending only on temperature, and $N_{O_{2}}$ is the concentration of oxygen. The lithium diffusion coefficient is expressed as:

(3) $$D\left(x\right)=\frac{D_{0}}{2}\left[\frac{N_{Li}\left(x\right)-N_{O_{2}}+\frac{1}{k}}{\sqrt{{\left(N_{Li}\left(x\right)-N_{O_{2}}-\frac{1}{k}\right)}^{2}+\frac{4N_{Li}\left(x\right)}{k}}}+1\right]$$

Thus, in conditions of formation of Li-O complexes, the coefficient of lithium diffusion is the function of $N_{O_{2}}$ and $N_{Li}$ concentration and equilibrium coefficient, *k*(*T*).

Under the coordinate dependence of *D*(*x*), the diffusion equation will be:

(4) $$\frac{\partial }{\partial t}N\left(x,t\right)=\frac{\partial }{\partial t}\left(D\left(x\right)\frac{\partial N\left(x,t\right)}{\partial x}\right)$$

A direct solution of Equation (4) with allowance for (3) is very difficult. However, there is a class of boundary conditions for (4) under which the diffusion equation with a coordinate-dependent diffusion coefficient admits an analytical solution into the Boltzmann-Matano substitution of the variables, $\eta =\frac{x}{\sqrt{t}}$. The solution obtained under this condition is called “self-similar”. The indicated class of boundary conditions relates diffusion from a continuous source of lithium atoms from both flat sides of the Si, with boundary conditions: *N*(*x*, 0) = 0, *N*(0, *t*) = *N(L, t*) = *N_S_*. In this case, the final form of the equation will be as follows:

(5) $$\frac{N\left(\eta \right)}{N_{S}}=1-\frac{\int_{0}^{\eta }\frac{1}{D}exp\left(-\frac{1}{2}\int_{0}^{\eta }\frac{1}{D}\eta^{\prime }d\eta^{\prime }\right)d\eta }{\int_{0}^{\infty }\frac{1}{D}exp\left(-\frac{1}{2}\int_{0}^{\eta }\frac{1}{D}\eta^{\prime }d\eta^{\prime }\right)d\eta }$$

If the diffusion time is constant (*t* = const), then in the initial variables, “*x*” and “*t*”, the Equation (5) will be:

(6) $$\frac{N\left(x,t\right)}{N_{S}}=1-\frac{\int_{0}^{x}\frac{1}{D}exp\left(-\frac{1}{2t}\int_{0}^{x}\frac{1}{D}x^{\prime }dx^{\prime }\right)dx}{\int_{0}^{\infty }\frac{1}{D}exp\left(-\frac{1}{2t}\int_{0}^{x}\frac{1}{D}x^{\prime }dx^{\prime }\right)dx}$$

It is easy to check that if *D* = const in (6), then we can obtain:

(7) $$\frac{N\left(x,t\right)}{N_{S}}=1-\frac{\int_{0}^{x}exp\left(-\frac{x^{2}}{4Dt}\right)dx}{\int_{0}^{\infty }\frac{1}{D}exp\left(-\frac{x^{2}}{4Dt}\right)dx}=1-erfc\left(\frac{x}{2\sqrt{Dt}}\right)=erfc\left(\frac{x}{2\sqrt{Dt}}\right)$$

Note that in (6), the diffusion coefficient depends on the coordinate, because it is a function of concentration:

(8) D=D(N(x,T))=Ψ(x)

However, there is no true coordinate dependence of the *D*(*x*) due to the isotropy of the medium. Calculation of the diffusion profile of (6) can be carried out with the help of the computer iterative method, because the integration function of (6) on the right side depends on calculating variables. The standard method for this is to expand *D*(*N*) in a power series:

(9) $$D\left[N\left(x,t\right)\right]=D_{0}+\lambda_{0}\cdot N\left(x,t\right)+\lambda_{1}\cdot N_{1}\left(x,t\right)+\ldots$$

in sequential computation of *N*(*x*, *t*) in the first (*D*_0_ = const), second, etc., approximations.

Special interest attracts consideration of a particular case, when $N_{\mathrm{Li}}<<N_{O_{2}}$, because these conditions are usually well-satisfied for silicon, grown by the method of Czochralski ($N_{O_{2}}$ ≈ 10^17^–10^18^ cm^−3^). In this case, in (3), the $N_{\mathrm{Li}}$ in comparison with $N_{O_{2}}\mathrm{can}\;\mathrm{be}\;\mathrm{neglected}\;$. Then,

(10) $$D=\frac{D_{0}}{1+k\left(T\right)N_{O_{2}}}$$

**Figure 2:**
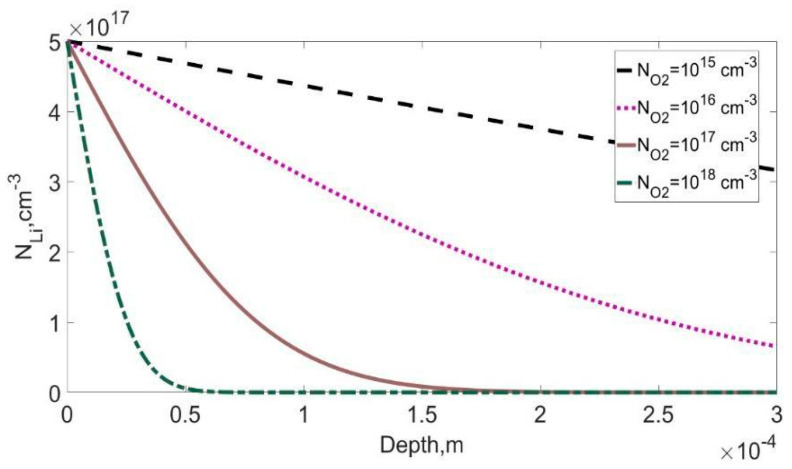
The diffusion profile of lithium into silicon at degrees of oxygen concentration from 10^15^ to 10^18^, at a temperature of T = 420 °C.

**Figure 3:**
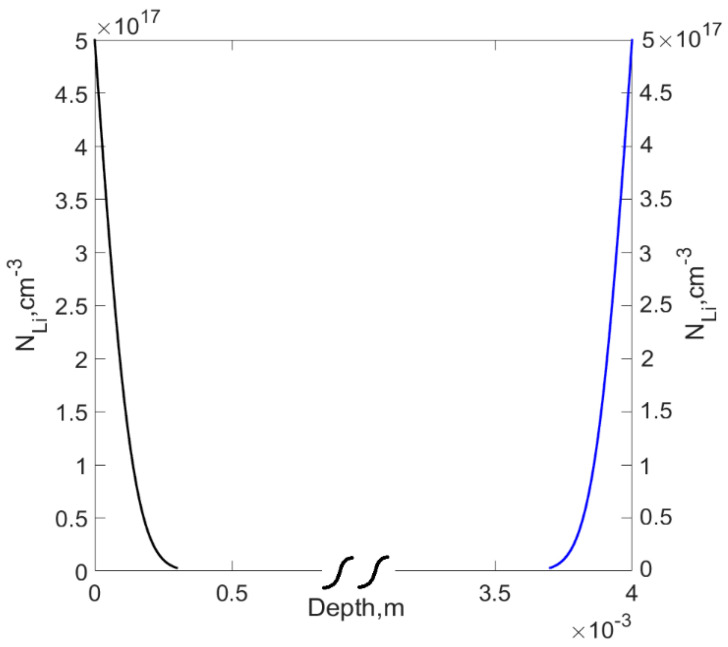
Double-sided diffusion profile of Li atoms into Si, at a temperature T = 420 °C, to the depth of 300 µm from both sides of the Si crystal.

Under intensive formation of Li-O complexes, the value of $k\left(T\right)\cdot N_{O_{2}}$ can be significantly high, which can lead to the decrease of the effective diffusion coefficient (see Figure 2). On the other hand, the absence of a coordinate dependence of the diffusion coefficient of lithium in the form of dependence (10) can make it possible to use the well-known solution of the diffusion Equation (7) for this case (see Figure 3).

It must be noted that the choice of the temperature–time regimes of diffusion of Li into Si must be performed under the conditions of thermo-defect formation. The interval of the temperature 300–500 °C, usually used for Li diffusion in Si(Li) p-i-n detectors, consists of a critical temperature of 450 °C, at which there is an intense generation of donor-type thermal defects [26].

According to the existing lithium-drift technology of Si(Li) p-i-n+ detectors, to obtain the desired lithium diffusion profile, the crystal is rapidly cooled in order to prevent “blurring” of the lithium distribution during the cooling process. However, the cooling rates achieved in this case (10^2^–10^3^) deg/s also lead to the formation of quenching-type thermal defects, which subsequently will negatively affect the characteristics [27].

## 4. Drift of Li-Ions into Si

**Figure 4:**
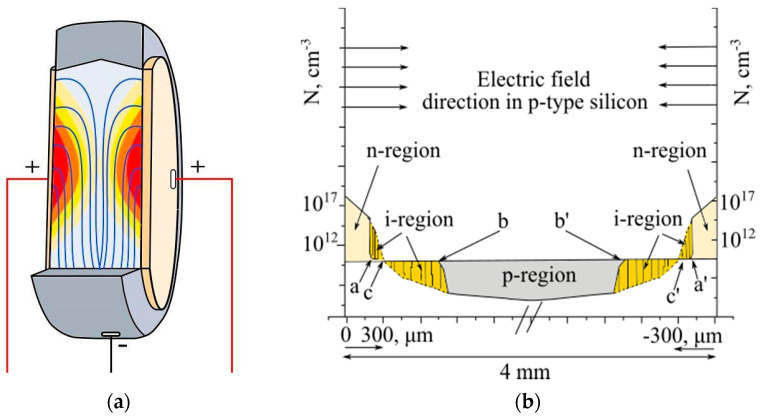
(**a**) Electric field distribution in the detector during the drift process, and (**b**) distribution of lithium-ions through the cross-section profile of the silicon crystal during the double-sided drift process.

We made an assumption of the physical description of the double-sided compensation of lithium-ions in silicon based on Pell’s theory of compensation. Accordingly, a layer of lithium was deposited on a flat circular plate, which is a donor with concentration *N*_0_ = 5 × 10^17^, by diffusion in vacuum at a depth of 300 μm from both surfaces of the plate for time *t* = 3–4 min at a temperature of T = 380–450 °C, until the p-n junction is formed at the positions x = c. The distribution of lithium is symmetric at the center of two flat surfaces of the crystal. According to the sufficiently large thickness of the crystal, various boundary effects that contribute to the internal interaction of the particles can be neglected, and it is possible to consider the particle distribution as a superposition of two independent functions (see Figure 4a,b).

We consider the simple model of drift of lithium-ions in a silicon crystal. In the general case, the movement of charged particles directly depends on the change in the electric field. In [13], using the Poisson equation, the dependence of the electric field on the depth of action can be shown as follows:

(11) $$\frac{dE\left(x\right)}{dx}=\frac{q}{\varepsilon \varepsilon_{0}}\left[N_{L}\left(x\right)-N_{A}\left(x\right)+p\left(x\right)-n\left(x\right)\right]$$

where *E* is the electric field strength in the semiconductor, *x* is the depth, *q* is the charge of the electron, *ε* is the dielectric constant of silicon, *ε*_0_ = 8.85∙10^−12^ F/m, *N_L_*(*x*) is the lithium concentration, *N_A_*(*x*) is the concentration of acceptors in semiconductors, *n*(*x*) is the concentration of free electrons and *p*(*x*) is the concentration of free holes.

(12) $$f\left(x\right)=D\frac{\partial N_{L}}{\partial x}+\mu N_{L}E$$

Then, it is possible to determine the concentration of lithium at any time depending on the depth using the continuity equation and flux density (12) of lithium, as shown in (13):

(13) $$\frac{\partial N_{L}}{\partial t}=D\frac{\partial^{2}N_{L}}{\partial x^{2}}+\mu \frac{\partial }{\partial x}\left(N_{L}-E\right)$$

where *μ* is the lithium-ion mobility in a semiconductor material. The solution of the system of Equations (11) and (13) requires simplifications, which are provided in [1]. In particular, if we assume that there is a complete compensation of lithium-ions, *N_L_*(*x*) − *N_A_*(*x*) = 0, then Equation (11) will be simplified to:

(14) $$\frac{dE\left(x\right)}{dx}=\frac{q}{\varepsilon \varepsilon_{0}}\left[p\left(x\right)-n\left(x\right)\right]$$

In [1], the dependence of non-recombined free charge carriers in a crystal is provided in the form:

(15) $$p\left(x\right)-n\left(x\right)=n_{i}\left(\frac{1}{\mu_{p}}+\frac{1}{\mu_{n}}\right)\frac{x}{2\tau_{r}E}-n_{i}\frac{W}{2\mu_{n}\tau_{r}E}$$

Substituting (15) into (14) and solving the resulting differential equation, we obtain the dependence of the electric field strength on depth in an explicit form:

(16) $$E\left(x\right)=\sqrt{\frac{2\left(E_{0}{}^{2}-\frac{\left(\frac{n_{i}}{\mu_{p}}+\frac{n_{i}}{\mu_{n}}\right)\frac{q}{\varepsilon \varepsilon_{0}}x^{2}}{2}-\frac{1}{2\tau_{r}}n_{i}\frac{W}{2\mu_{n}\tau_{r}}\frac{q}{\varepsilon \varepsilon_{0}}\right)}{\frac{1}{2\tau_{r}}}}$$

**Figure 5:**
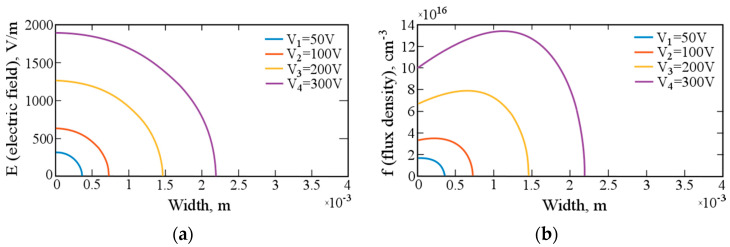
(**a**) Change in the electric field during the drift of lithium-ions at different applied voltages. (**b**) Change in the flux density of lithium-ions in the crystal volume at different applied voltages.

Figure 5a shows the dependence of the electric field strength on the depth for a different initial electric field applied to the crystal. Figure 5b shows the graphs of the change in the lithium-ion flux density depending on the voltage applied to the crystal. As can be seen from the graphs, with increasing depth, the electric field rapidly decreases and the ion drift velocity also decreases, which is reflected in the change in the lithium-ion flux density.

**Figure 6:**
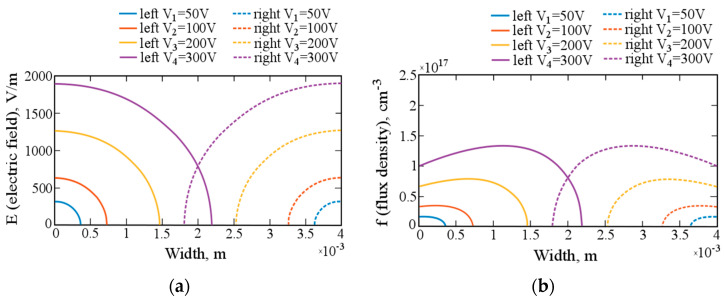
(**a**) Electric field distribution in the cross-section of the crystal during the double-sided drift process. (**b**) The flux density of lithium-ions in the crystal during the double-sided drift process.

In the case of double-sided drift of lithium-ions into a silicon monocrystal, it is necessary to jointly solve Equation (14) to obtain the dependence of the electric field on the depth. Figure 6a shows the electric fields in a crystal for double-sided drift. With an increase in the voltage applied to the crystal, the depth of action of the field increases, and, consequently, the drift velocity and the flux of carriers of charge also increase.

Figure 6b shows the change in the flux density of lithium-ions during the double-sided drift process. The flux density increases at the beginning of the process with some inertia and rapidly decreases due to a decrease in the electric field.

The obtained dependences, although they characterize the behavior of lithium-ions, due to the introduced assumptions, do not fully describe the drift process. With incomplete compensation of lithium-ions, the expression *N_L_*(*x*) − *N_A_*(*x*) ≠ 0, as shown in [11], should be rewritten as follows:

(17) $$N_{L}\left(x\right)-N_{A}=\frac{\varepsilon \varepsilon_{0}Vg^{2}}{q{\left(1+gx\right)}^{2}lnln\left(1+gL\right)}$$

where *V* is the voltage applied to the crystal, *L* is the compensating region width, and *g* is the acceptor gradient.

We introduce a time dependence using the assumption that the drift velocity, υ, directly depends on the electric field [28]:

(18) $$v=\mu_{L}E$$

where *μ_L_* is the lithium-ion mobility in a crystal. Then, the expressions for the *x* coordinate, expressed in terms of the time, *t,* can be expressed in a linear way:

(19) $$x=vt=\mu_{L}Et$$

Then, we finally obtain (20):

(20) $$\frac{dE\left(t\right)}{dt}=\frac{q}{\varepsilon \varepsilon_{0}}\left[\frac{\varepsilon \varepsilon_{0}Vg^{2}}{q{\left(1+g\mu_{L}Et\right)}^{2}lnln\left(1+gL\right)}+n_{i}\left(\frac{1}{\mu_{p}}+\frac{1}{\mu_{n}}\right)\frac{\mu_{L}Et}{2\tau_{r}E}-n_{i}\frac{W}{2\mu_{n}\tau_{r}E}\right]$$

**Figure 7:**
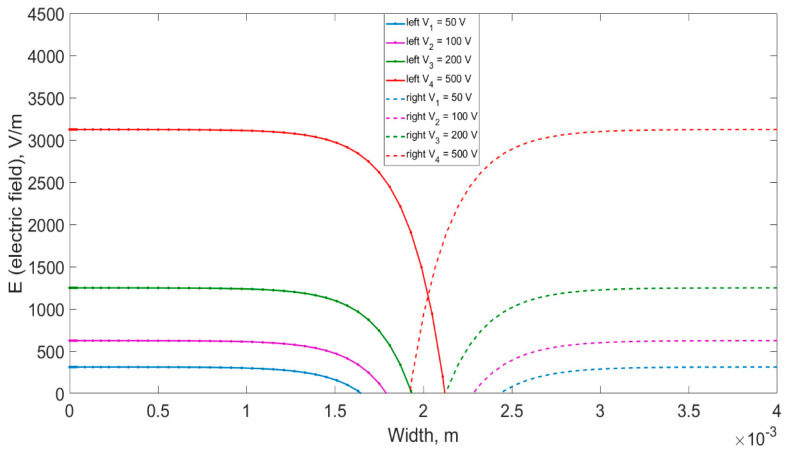
Electric field distribution in the cross-section of the crystal during the double-sided drift process, taking into account the presence of uncompensated lithium-ions.

The solution to this equation in numerical form is shown in Figure 7 for the double-sided drift of lithium-ions.

**Figure 8:**
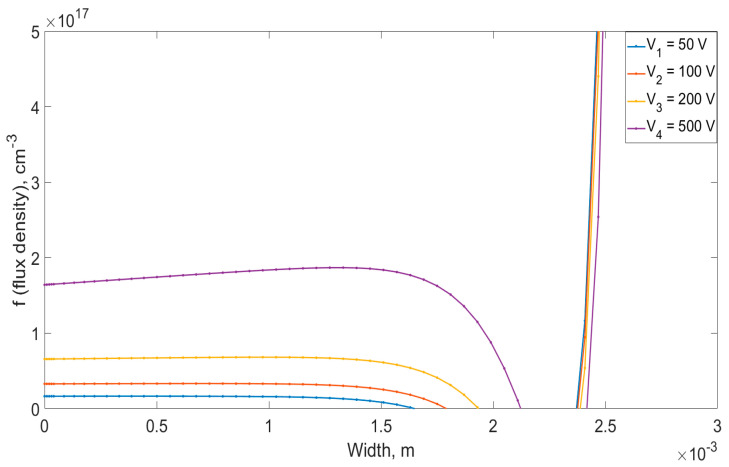
The flux density of lithium-ions in a crystal during the double-sided drift process, taking into account the presence of uncompensated lithium-ions.

Figure 8 shows the dependence of the lithium-ion flux density, during the double-sided drift process, taking into account the presence of uncompensated ions in the crystal volume. This figure shows the ion flow for only one side. For the second side of the detector, the graph behaves similarly. The shapes of the plots obtained are also similar to those in Figure 8, however, due to the fact that the flow of lithium-ions meets the counter flow of ions from the other side, the concentration of particles increases sharply, which leads to a sharp increase in the flow, which is illustrated in Figure 8.

Now, we take into account the effect of the counter flow of lithium-ions in the crystal, which is expressed in the creation of a space charge region. This space charge prevents the build-up of the electric field on the other side. As a result, it is necessary to introduce an additional term into Equation (20), expressing the effect of the counter electric field. Since the macroscopic velocity of lithium-ions is low, the counter field can be expressed using the Poisson equation. Then, Equation (20) will take the form:

(21) $$\frac{dE\left(t\right)}{dt}=\frac{q}{\varepsilon \varepsilon_{0}}\left[\frac{\varepsilon \varepsilon_{0}Vg^{2}}{q{\left(1+g\mu_{L}Et\right)}^{2}lnln\left(1+gL\right)}+n_{i}\left(\frac{1}{\mu_{p}}+\frac{1}{\mu_{n}}\right)\frac{\mu_{L}Et}{2\tau_{r}E}-n_{i}\frac{W}{2\mu_{n}\tau_{r}E}\right]-\frac{dE_{c}\left(t\right)}{dt}$$

where *E_C_* is the electric field of the counter flow of lithium-ions.

**Figure 9:**
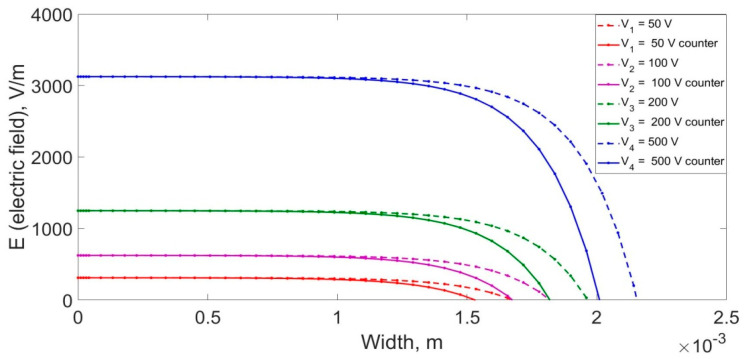
The impact of the counter flow of lithium-ions on the propagation of the electric field in the crystal during various voltage applications.

Figure 9 shows the effect of a counter flow of lithium-ions on the propagation of an electric field in a crystal. Solid lines show a rapid decrease in the electric field under the influence of the counter electric field of lithium-ions of the counter flow.

## 5. Electrical Characteristics of the Si(Li) p-i-n Structure

The degree of compensation in lithium-drift technology of p-i-n structures depends on the temperature-field regimes [29]. By choosing the optimum values of the reverse bias voltage and the drift temperature, it is possible to achieve the maximum degree of compensation, which is characterized by the volume charge in the considered point c and c′, shown in Figure 4, of the compensated i-region. Table 1 shows the experimental data on the mode of reverse bias voltage and temperature, which gradually increase with the expansion of the compensated i-region.

Table 1 shows the experimental regimes of the reverse bias voltage and temperature, which gradually increase with the expansion of the compensated i-region. The relative thickness of the high-resistivity layers sharply decreases with increasing temperature in the interval 55–90 °C, and then is fixed at T = 110 °C. The drift regime at T = 110 °C and a voltage U = 300 V is characterized by a low reverse current, and a sufficiently high temperature leads to resorption of precipitates [30], i.e., it can be assumed that the micro-inhomogeneities of the composition are smoothed out. Thus, the most suitable regime for drift is at the temperature T = 110 °C and the voltage U = 300 V.

In [31], the conventional, one-sided drift technology, for large-sized detectors with a thickness of 3 mm, was reported. Table 1 describes experimental data of double-sided drift technology. As can be seen from Table 1, in the proposed method, the drift time is considerably shorter than in [31], when months of work are taken to obtain a structure with a thickness W ≥ 4 mm.

**Figure 10:**
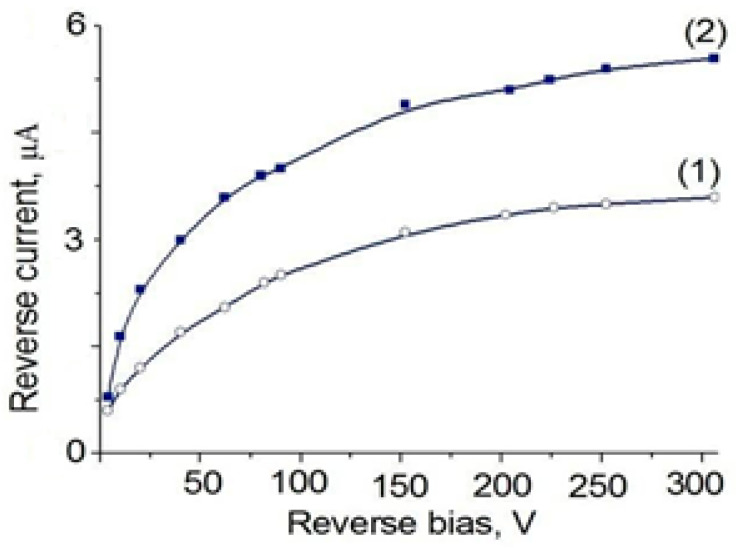
The inverse branch of I–V of the Si(Li) p-i-n structure, grown by the Czochralski process (line 1) and floating-zone method (line 2).

After preparation of a Si(Li) p-i-n detector’s structure, the I–V characteristics during application of reverse bias voltage were investigated (see Figure 10). Here, I–V for silicon wafers obtained from high-resistance silicon grown by the float-zone method (line 2) and low-resistance silicon grown by the Czochralski process (line 1) were compared.

In industrial detectors of large diameters obtained by the traditional method, fluctuations of the leakage current and charge collection time are noticed, thereby deteriorating the energy characteristics of the detector [32,33]. Small values of leakage current and a smooth current–voltage characteristic (shown in Figure 10) proves a uniform distribution of charge carriers, in other words, uniform compensation, throughout the volume of the crystal [34,35]. It is due to the fact that in double-sided drift, the path of the carriers is reduced by two and the drift time by four times. The small reversed current of the low-resistance silicon grown by the Czochralski method associated with high exploitation characteristics has the advantage of manufacturing lithium drift detectors with large volume, compared with crystals obtained by the float-zone method. However, the drift of lithium-ions into silicon, grown by the Czochralski method, has significant features, due to the high content of impurities in its structure. This can lead to self-heating of the crystals during the drift process. Taking into account these features, we have come up with optimal temperature regimes of the stepped drift of lithium-ions in silicon.

**Figure 11:**
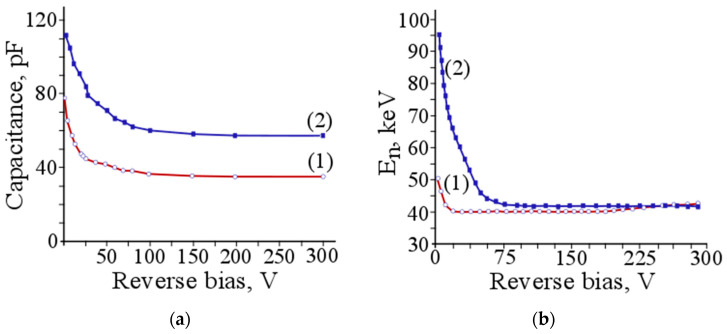
(**a**) Capacity–voltage and (**b**) noise–voltage characteristics of the Si(Li) p-i-n structure, grown by the Czochralski process (1) and the float-zone method (2).

The same samples, which were used to investigate the I–V characteristics, were used to study capacity–voltage and noise–voltage characteristics. In Figure 11a, the capacity–voltage characteristics of the Si(Li) p-i-n structure are shown. The capacitance of the Si(Li) p-i-n structure is directly connected with the thickness of the depletion layer and with a specific resistance of the initial material. Therefore, by measuring this, it is possible to identify the specific resistance of the compensated area of silicon in the prepared structure and predict the values of maximum energy of the charged particle, under the conditions of its total absorption in the depletion layer.

## 6. Conclusions

The paper considered the manufacturing process of large-sized p-i-n structured Si(Li) radiation detectors by double-sided diffusion and drift technology.

A theoretical description of double-sided diffusion of Li atoms into monocrystalline silicon was considered, taking into account the presence of a high concentration of Li-O complexes in the volume of the crystal and in the regimes of temperature T = 420 °C to the depth of *h_Li_* = (300 ± 10) µm, for *t* = 3 min.

The theoretical model of the double-sided drift of lithium-ions into the crystal volume was considered, taking into account regimes of the double-sided drift, a synchronous stepwise increase in temperature from 55 to 110 °C and a reverse bias voltage from 70 to 300 V, which creates an external electric field from both flat ends of the crystal. The proposed theoretical model is based on the homogeneity of acceptor atoms in a crystal, where there can be a dynamic equilibrium in which lithium-ions move uniformly in the depleted region, producing no net charge in the lithium concentration, except in the boundary.

At the end of this work, we presented the experimental regimes of providing double-sided drift of Li-ions into Si. Based on this, it can be argued that the proposed method reduced the drift time by up to 4 times compared with the traditional one-sided drift method. This helps to avoid the following main problems in the manufacture of large Si(Li) detectors: the formation of a defect in the body of the crystal due to the high temperature and long heating time, inhomogeneous distribution of lithium-ions over the entire volume of the silicon crystal and spending too long on manufacturing the detector.

Additionally, according to the experimental electrophysical characteristics of the detectors obtained by the proposed method, it can be argued that these detectors have a low reverse current, thereby proving a more uniform distribution of lithium-ions throughout the entire volume of the crystal. Therefore, the small reverse current is the main indicator of the high efficiency of Si(Li) detectors.

For semiconductor detectors, which operate at room temperature, used for low-noise installations, these results are very important, since it opens up new possibilities for improving the registration efficiency due to the energy resolution and large dimensions of the detectors.

## Figures and Tables

**Table 1:** Temperature and reverse bias voltage regimes.

Regimes	Temperature, °C	Voltage, V	Time, Hour
1	55	70	6
2	70	100	16
3	80	130	24
4	110	300	28
5	50	90	10

## Data Availability

Data sharing is not applicable to this article.
